# Optical analysis of CH_3_NH_3_Sn_*x*_Pb_1–*x*_I_3_ absorbers: a roadmap for perovskite-on-perovskite tandem solar cells†
†Electronic supplementary information (ESI) available. See DOI: 10.1039/c6ta04840d



**DOI:** 10.1039/c6ta04840d

**Published:** 2016-06-29

**Authors:** Miguel Anaya, Juan P. Correa-Baena, Gabriel Lozano, Michael Saliba, Pablo Anguita, Bart Roose, Antonio Abate, Ullrich Steiner, Michael Grätzel, Mauricio E. Calvo, Anders Hagfeldt, Hernán Míguez

**Affiliations:** a Institute of Materials Science of Seville , Spanish National Research Council-University of Seville , Américo Vespucio 49 , 41092 , Seville , Spain . Email: h.miguez@csic.es; b Laboratory for Photomolecular Science , Institute of Chemical Sciences and Engineering , Ecole Polytechnique Fédérale de Lausanne , CH-1015-Lausanne , Switzerland . Email: juan.correa@epfl.ch; c Laboratory for Photonics and Interfaces , Institute of Chemical Sciences and Engineering , Ecole Polytechnique Fédérale de Lausanne , CH-1015-Lausanne , Switzerland; d Adolphe Merkle Institute , Chemin des Verdiers 4 , CH-1700 Fribourg , Switzerland

## Abstract

Organic–inorganic perovskite structures in which lead is substituted by tin are exceptional candidates for broadband light absorption. Herein we present a thorough analysis of the optical properties of CH_3_NH_3_Sn_*x*_Pb_1–*x*_I_3_ films, providing the field with definitive insights about the possibilities of these materials for perovskite solar cells of superior efficiency. We report a user's guide based on the first set of optical constants obtained for a series of tin/lead perovskite films, which was only possible to measure due to the preparation of optical quality thin layers. According to the Shockley–Queisser theory, CH_3_NH_3_Sn_*x*_Pb_1–*x*_I_3_ compounds promise a substantial enhancement of both short circuit photocurrent and power conversion efficiency in single junction solar cells. Moreover, we propose a novel tandem architecture design in which both top and bottom cells are made of perovskite absorbers. Our calculations indicate that such perovskite-on-perovskite tandem devices could reach efficiencies over 35%. Our analysis serves to establish the first roadmap for this type of cells based on actual optical characterization data. We foresee that this study will encourage the research on novel near-infrared perovskite materials for photovoltaic applications, which may have implications in the rapidly emerging field of tandem devices.

## Abstract 2

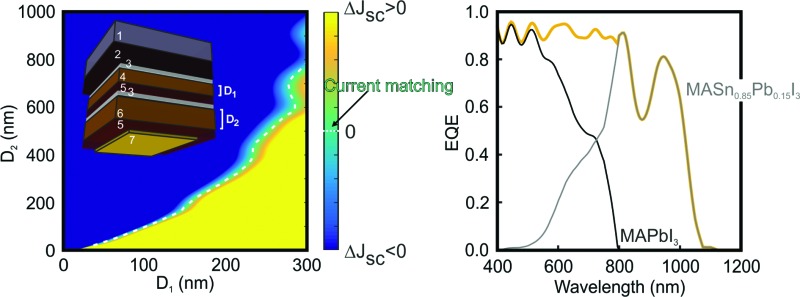
We propose a novel tandem architecture design in which both top and bottom cells contain perovskite absorbers.

In the last few years methylammonium lead iodide, a well-known compound with a perovskite crystal structure^1–3^ has come into light as a unique material for solar power and optoelectronic applications.^4–7^ Indeed, CH_3_NH_3_PbI_3_ and alternative APbX_3_ perovskites yield unprecedented results in solar energy conversion devices^8–12^ due to their large extinction coefficient, low exciton binding energy, and large charge diffusion length.^13,14^ Since the performance of standard lead-based perovskite devices is reaching the theoretical limit,^15,16^ nowadays, most research efforts pursue to unravel the electrical and photophysical properties of perovskite materials,^13,17,18^ in order to achieve devices with stabilised efficiencies,^19–21^ and long-term durability.^22–24^

A promising but scarcely explored route to achieve further efficient devices consists in the development of novel perovskite light harvesters.^25^ In particular, those in which lead is substituted by other metals,^26^ especially tin, give rise to narrower bandgap energies, which results in a better harnessing of the incident sunlight.^27–35^ However, limited film quality and fast oxidation of Sn^2+^ to Sn^4+^, which induces self-doping that turns the semiconducting perovskite into a conducting one,^36^ hamper the power conversion efficiencies (PCEs) of actual devices that never surpass few percents.^28–35^ Nevertheless, the interest in them is still enormous, as this particular type of perovskite is the only one that permits extending the bandgap to the near-infrared (NIR) and hence reaching the Shockley–Queisser (SQ) limit. A widely explored route to surpass the SQ limit consists of the combination of two or more single junction devices into a tandem system to harvest a larger fraction of the sunlight. So far, efforts are intensifying to demonstrate double-junction devices in which APbX_3_ perovskites and CIGS or Si materials are employed as top and bottom cells, respectively.^37–40^ Alternatively, tin–lead perovskites of tuneable bandgap offer a complete new range of possibilities to develop solution processed absorbers to be integrated into devices with tandem configuration of higher efficiency. However, a thorough analysis on the implications of such tin–lead perovskites for photovoltaic applications is still missing.

In this paper we employ a novel synthesis and deposition route to obtain smooth and pinhole-free CH_3_NH_3_Sn_*x*_Pb_1–*x*_I_3_ films with high optical quality by solution processed techniques. This allows performing an in-depth optical characterization of the films, and extracting, for the first time, the complex refractive index of the different perovskite compositions based on the Sn/Pb alloys. We use a rigorous theoretical model, based on the transfer matrix formalism,^41^ to predict the optical behaviour of a solar device integrating these materials and to estimate the maximum short circuit photocurrent (*J*_sc_) values expected for each Sn/Pb ratio. The combination of bandgap engineering and high optical quality achieved allow us to propose a perovskite-on-perovskite tandem solar cell architecture in which CH_3_NH_3_PbI_3_ and CH_3_NH_3_Sn_0.85_Pb_0.15_I_3_ perovskite films are employed as active layers for the top and bottom cell, respectively. This design promises to be of great importance for the immediate future of this rapidly growing field.

Perovskite materials with changing Sn and Pb compositions in CH_3_NH_3_Sn_*x*_Pb_1–*x*_I_3_, where *x* is varied from 0 to 1 (otherwise denoted throughout the text as 0% and 100%, respectively), are prepared by taking different volumes of two stock solutions of Pb and Sn perovskite precursors. Although full details are provided in the Methods section, it is worth mentioning that optical quality films were only obtained by means of a combination of precursor solvent engineering and anti-solvent deposition method, which induces fast crystallization of the mixed perovskite. Fig. 1a–c show the cross-sectional SEM images of the perovskite films with 0%, 50% and 100% content of Sn. Our synthesis route drives to layers that present smooth thickness variations, which are small enough to consider the perovskite–air interface plane-parallel for the impinging light. From top view SEM images (see Fig. 1d–f), it can be observed that crystalline grains are densely packed forming continuous films atop the mesoporous TiO_2_ layer, presenting no degradation at room temperature (see Fig. S1†). The crystal structures of compositions varying from 0% to 100% Sn were analysed by XRD (Fig. 1g). The gradual shift of the two dominant peaks appearing in all the composites, 2*θ* = 14° and 28.5°, is monitored as seen in Fig. 1h and i. This phenomenon has been shown by Hao *et al.*, where the transition from the tetragonal to the cubic phase is explained by the distortion of the relative positions of the octahedra due to the randomly occupied Sn or Pb.^32,34,42^ Our deposition method induces the formation of large crystals, key for improving charge carrier transport in PSCs and yielding optimal photovoltaic performance in pure Pb perovskite solar cells.^15,43^

**Fig. 1:**
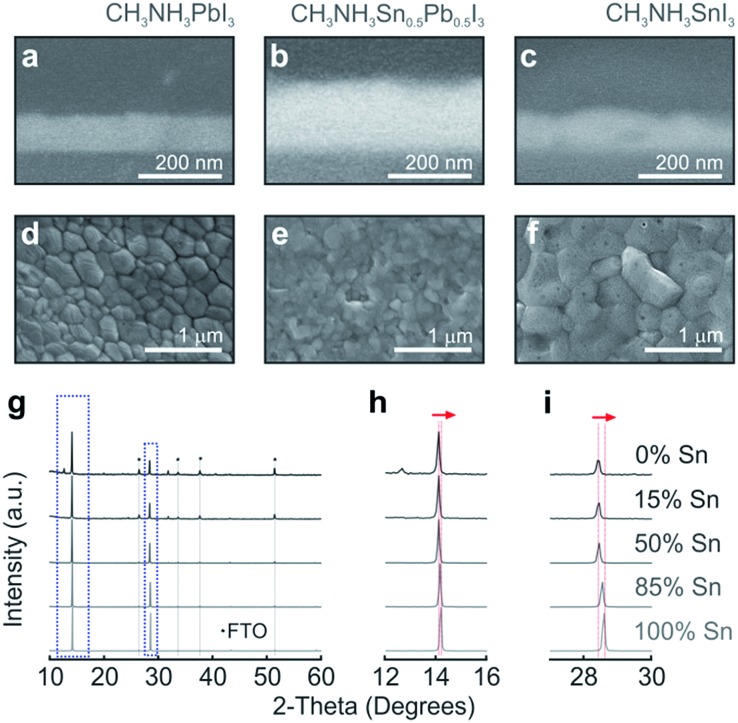
Cross-sectional (a–c) and top view (d–f) SEM images of the perovskite materials with different compositions as deposited on a glass substrate for optical analysis. Perovskite layers have been shaded with colours for the sake of clarity. (g) XRD diagrams corresponding to films in which the Sn/Pb ratio is gradually varied. Details of the 14° (h) and 28.5° (i) diffraction peaks.

The pin-hole free, dense and planar character of the films prepared, which is preserved with time (see Fig. S1 and S2 of the ESI†), allows us to conduct a thorough study on the optical properties of perovskite films with different metal compositions, *e.g.* CH_3_NH_3_PbI_3_, CH_3_NH_3_Sn_0.15_Pb_0.85_I_3_, CH_3_NH_3_Sn_0.5_Pb_0.5_I_3_, CH_3_NH_3_Sn_0.85_Pb_0.15_I_3_ and CH_3_NH_3_SnI_3_. Our spectroscopic analysis reveals the high optical quality of the perovskite films, which do not suffer from the effect of diffuse scattering (see Fig. S3 of the ESI†). Absorptance measurements of the metal based mixed perovskite films are displayed in Fig. S4,† indicating a bandgap evolution already discussed elsewhere.^44^ The small fraction of the incident light absorbed at wavelengths larger than the bandgap discards the existence of intraband states, demonstrating the low density of defects present in the fabricated films.

Our optical measurements enable the analysis of the gradual change of the optical constants of the composites as the amount of tin increases at the expense of lead. Kramers–Kronig consistent real and imaginary parts of the complex refractive index of perovskite thin films with different compositions are displayed in Fig. 2a–e (see Fig. S5 of the ESI† for the complex dielectric permittivity). All fitting parameters can be found in Table S1 of the ESI.† The perovskite materials under analysis feature complex refractive indexes of similar order of magnitude regardless of the specific perovskite composition, implying that the Sn/Pb composites should be as efficient light harvesters as the standard Pb based perovskite. Fair agreement between theory (dashed lines) and experiment (solid lines) is found for the reflectance and transmittance spectra obtained for the different Sn/Pb perovskites, as displayed in Fig. 2f–j.

**Fig. 2:**
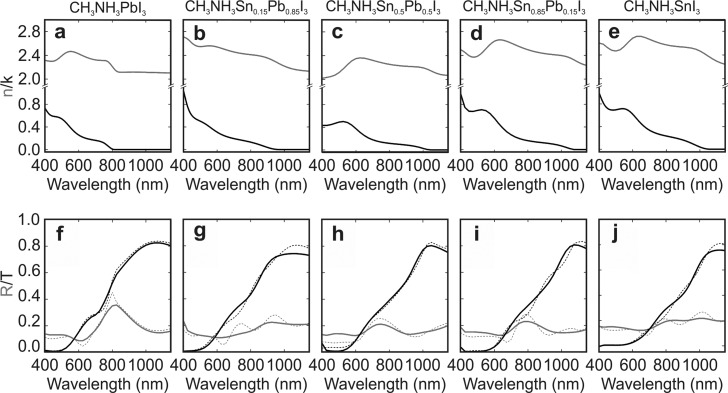
(a–e) Real (grey) and imaginary (black) parts of the complex refractive index of the mixed metal perovskite structures herein studied. (f–j) Experimental (dashed) and calculated (solid) spectra of the reflectance (grey) and transmittance (black) corresponding to the different films.

The possibility to tune the bandgap of CH_3_NH_3_Sn_*x*_Pb_1–*x*_I_3_, from 1.60 eV for *x* = 0 to 1.17 eV for *x* = 0.85 (see Fig. 3a), brings the opportunity to develop the ideal absorber to maximize solar conversion efficiency. Fig. 3b displays the maximum theoretical *J*_sc_ and PCE that may be achieved as a function of the electronic energy gap (*E*_g_) of the active material according to the SQ theory (see Methods section).^45^ As it is well known, the ideal *J*_sc_ increases as the *E*_g_ is reduced since a larger fraction of the incident sunlight is harvested by the absorbing material. In contrast, charge recombination increases with the reduction of *E*_g_. As a consequence, a limiting PCE over 33% could be attained for *E*_g_ = 1.14 eV or *E*_g_ = 1.34 eV, considering an idealized system without additional losses, such as parasitic absorption or non-radiative recombination.

**Fig. 3:**
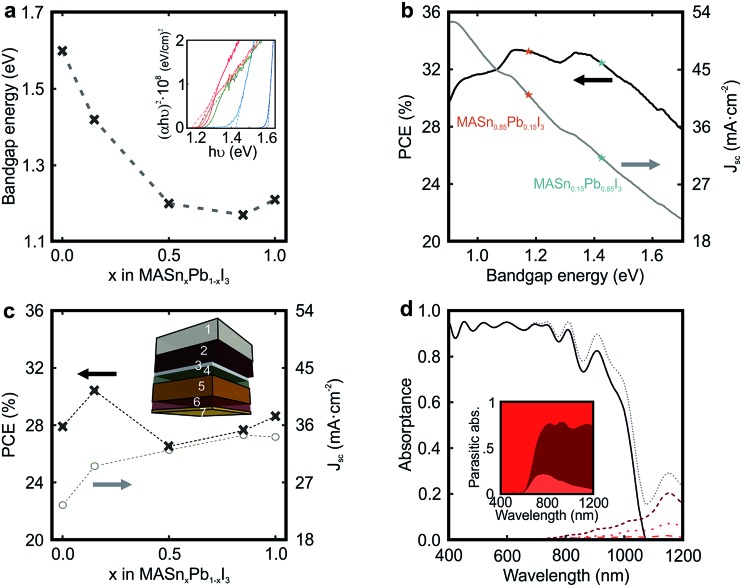
(a) Bandgap energy of the different mixed metal perovskite compositions *versus* the amount of Sn in the structure. The dashed grey line is only a guide for the eye. Inset shows the Tauc plots employed to estimate the direct bandgap energy. (b) Ideal calculated short circuit photocurrent (*J*_sc_) and power conversion efficiency (PCE) as a function of the bandgap energy according to the Shockley–Queisser theory. (c) Calculated *J*_sc_ and PCE for simulated devices in which the percentage of Sn is increased at the expense of Pb. Inset shows the architecture of the simulated device: 1 mm of glass substrate (1), 650 nm of FTO layer (2), 50 nm of TiO_2_ compact layer (3), 85 nm of 50% porous TiO_2_ scaffold fully infiltrated by perovskite (4), 600 nm of perovskite capping layer (5), 240 nm of spiro-OMeTAD (6), and 50 nm of gold contact (7). (d) Spectral dependence of the fraction of light absorbed by the different layers comprising the cell: FTO (red dotted line), CH_3_NH_3_Sn_0.85_Pb_0.15_I_3_-based perovskite (black solid line), spiro-OMeTAD (brown short dashed line), and gold contact (orange dashed line). Inset shows details of the spectral dependence of the parasitic absorption in the FTO, spiro-OMeTAD and gold.

In order to evaluate the impact of the integration of CH_3_NH_3_Sn_*x*_Pb_1–*x*_I_3_ films in actual solar cells, we introduce their optical constants in a model previously used to describe the performance of PSCs.^41^ The optical constants of the materials employed to model the cell are available in Fig. S6 of the ESI.† By this means, we can estimate the fraction of incident sunlight absorbed by the perovskite in a standard cell, whose scheme is represented in the inset of Fig. 3c. We assume a 600 nm perovskite capping layer, as this value has been demonstrated to yield state of the art efficiencies in pure Pb-based devices.^15^ In Fig. 3c, we present the calculated *J*_sc_ for devices in which the Sn/Pb ratio is varied. See Fig. S7 of the ESI† for a detailed analysis on the influence of the capping layer thickness. Our model considers (i) parasitic losses associated with the different absorbing materials present in the cell (FTO, spiro-OMeTAD or gold), (ii) reflection losses at the entrance of the cell, and (iii) ideal charge transport and charge collection, which implies that the internal quantum efficiency equals the light harvesting efficiency (IQE(*λ*) = LHE(*λ*)). It can be observed that the maximum *J*_sc_ follows the evolution of *E*_g_ (see Fig. 3a), *i.e.* perovskites with lower bandgaps should give rise to higher photo-generated currents. In particular, for an ideal PSC in which CH_3_NH_3_Sn_0.85_Pb_0.15_I_3_ is used as the light harvester, a *J*_sc_ of 34 mA cm^–2^ is foreseen. Fig. 3c also displays the maximum PCE achievable for each Sn/Pb perovskite composition. Notice that *J*_sc_ measured from state-of-the-art pure lead devices is nearly ideal.^15^ However, record devices feature PCEs that do not meet the values estimated according to the SQ theory, *i.e.* PCE of 22.1% *vs.* 27.9%.^16^ In order to provide a more realistic estimation of the efficiency of the PSC, it would be necessary to consider a thorough model of its electrical behaviour, which is out of the scope of this study. Our analysis indicates that a relative PCE enhancement of ∼10% with respect to the standard Pb perovskite material may be attained just by substituting 15% Pb by Sn in the perovskite film precursor (PCE of 27.9% *vs.* 30.4%), due to a 25% increase in the *J*_sc_ (23.5 mA cm^–2^ *vs.* 29.5 mA cm^–2^) together with a more favorable *E*_g_. In contrast, our estimations performed using a real device architecture indicate that the film with 85% Sn content (*E*_g_ = 1.17 eV) would give rise to a PCE far from that predicted for the ideal one according to the SQ theory (PCE of 28% *vs.* 33% expected). In order to explain the origin of this behaviour, in Fig. 3d we plot the fraction of light that the different components of the CH_3_NH_3_Sn_0.85_Pb_0.15_I_3_-based PSC absorb. It can be observed that the ratio between parasitic absorption, caused by optical losses occurring in spiro-OMeTAD and FTO, and productive absorption increases significantly in the NIR spectral range as the bandgap of the perovskite is gradually reduced, which results in a *J*_sc_ that is far from the ideal according to the Shockley–Queisser theory (*J*_sc_ of 34 mA cm^–2^ *vs.* 41 mA cm^–2^ expected). In view of this result, it might be helpful, in order to minimize optical losses, to engineer novel hole transporting materials for cells operating in the NIR.

In order to prove the trends in photocurrent predicted by our optical model, we prepared devices of CH_3_NH_3_PbI_3_ and CH_3_NH_3_Sn_0.15_Pb_0.85_I_3_ perovskites. Full details are provided in the ESI: see Fig. S8 and S9.† Our experimental results indicate a substantial enhancement of the *J*_sc_ with respect to the standard Pb device, *J*_sc_ increasing from 19.5 mA cm^–2^ in the pure lead device to 24.8 mA cm^–2^ in the hybrid one, in fair agreement with our estimations. Despite the remarkable photocurrents attained, among the largest ever demonstrated for a perovskite material, and the relatively high voltages, comparable to those reported elsewhere,^30^ the performance of the Sn/Pb device is still modest due to the rather low fill factors (see Fig. S8 of the ESI†). Although the potential of Sn/Pb perovskite materials for solar cells seems undeniable, severe limitations of their chemical stability have prevented the observation of efficiencies close to the values herein predicted. A precise control of the oxygen exposure has proven to be critical to avoid the self-doping behaviour of Sn, which even in small quantities may be detrimental to achieve the expected performance. Oxygen levels as low as few parts per million can cause the oxidation of the Sn-based perovskite grains, which induces Sn^4+^ states which in turn increase charge carrier concentration. This translates into a degenerate semiconductor with metallic behaviour, inducing recombination centres, which has hampered so far the realization of efficient devices.^36^ Nevertheless, our measurements reveal the potential of Sn/Pb perovskite materials as light harvesters and open up a myriad of possibilities for optimization.

One of the most appealing properties of CH_3_NH_3_Sn_*x*_Pb_1–*x*_I_3_ perovskites is their narrow bandgap (1.17 < *E*_g_ < 1.60 eV) that opens the door to the realization of double-junction devices in which both top and bottom cells are based on perovskite absorbers. Indeed, a two-terminal monolithic architecture can be considered as a series circuit in which the current through the top and bottom cells is the same, and the voltage across the device is the sum of the voltages across each of the constituent cells. In order to optimize the performance of a tandem device, it is of utmost importance to find the configuration that gives rise to a similar fraction of the incident light harvested by the top and bottom cells: the so-called current-matching condition. We now want to provide the reader with a roadmap for the realistic design of perovskite-on-perovskite tandem devices. Although one precedent has been reported dealing with the combination of wide bandgap perovskites in order to achieve higher voltages,^46^ herein we propose a new architecture that, while providing a large voltage, is able to harvest a larger fraction of the incident light. As displayed in the inset of Fig. 4a, we consider a tandem solar cell with a planar configuration (see Fig. S10 of the ESI† for results concerning a mesostructured configuration). The active material chosen for the top cell is the standard CH_3_NH_3_PbI_3_ perovskite, which absorbs the incident light very efficiently up to 800 nm. Less energetic photons pass through the system and reach the CH_3_NH_3_Sn_0.85_Pb_0.15_I_3_ perovskite in the bottom cell, which harvests light up to *ca.* 1100 nm. Combined in a double-junction solar cell, the energy bandgaps (*E*_g_ = 1.6 eV and *E*_g_ = 1.17 eV) of these two materials are nearly optimized according to the SQ theory, similar to what occurs in CH_3_NH_3_PbI_3_/silicon tandem devices.^37,38,40^ SnO_2_ films are considered to act as electron selective layers that could be deposited by atomic layer deposition, for instance, for conformal low temperature deposition. This processing step is crucial, as it guarantees a proper band alignment of the perovskite materials with this electron selective layer, with the additional advantage of low temperature processing needed when dealing with perovskite materials.^38,47^

**Fig. 4:**
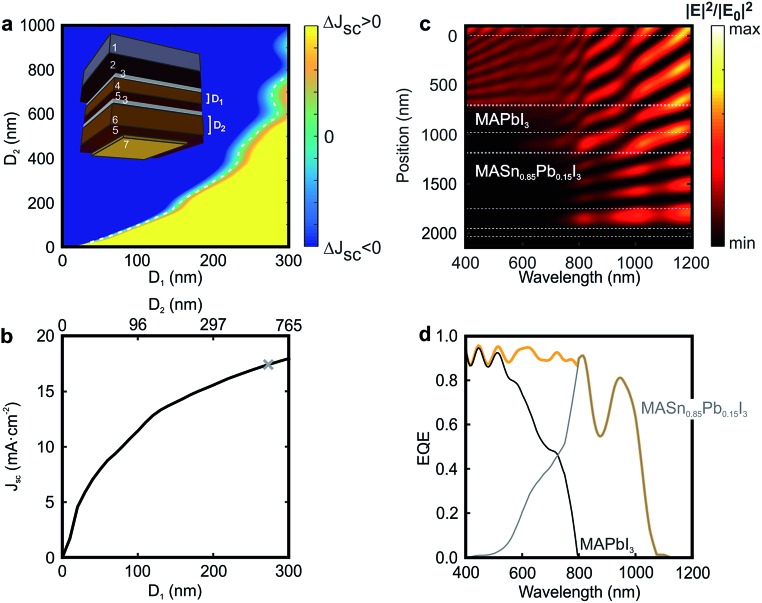
(a) Difference between short circuit currents extracted from the top and bottom cells composing a tandem device in which the CH_3_NH_3_PbI_3_ (*D*_1_) and CH_3_NH_3_Sn_0.85_Pb_0.15_I_3_ (*D*_2_) thicknesses are varied. The white dashed line indicates the thickness pairs for which the current matching is achieved. The inset shows the architecture of the simulated device: 1 mm of glass substrate (1), 650 nm of FTO layer (2), 50 nm of SnO_2_ compact layer (3), *D*_1_ nm of absorber CH_3_NH_3_PbI_3_ (4), 240 nm of spiro-OMeTAD (5), 50 nm of SnO_2_ compact layer (3), *D*_2_ nm of absorber CH_3_NH_3_Sn_0.85_Pb_0.15_I_3_ (6), 240 nm of spiro-OMeTAD (5) and 50 nm of gold contact (7). (b) Matched short circuit current values for each pair of *D*_1_–*D*_2_ thicknesses. The grey mark points out the configuration that is more deeply analysed in the next panels, in which *D*_1_ = 270 nm and *D*_2_ = 560 nm. (c) Calculated spatial (*y*-axis) and spectral (*x*-axis) distribution of the electric field intensity enhancement along the cross-section of the selected perovskite-on-perovskite tandem solar cell. White dashed lines specify the interfaces between the different layers. (d) Calculated external quantum efficiency corresponding to the top (black line) and bottom (grey line) cells. The orange line corresponds to the total external quantum efficiency for the complete tandem device.

We calculate the fraction of incident light that each of the perovskite materials absorbs using a model based on the transfer matrix formalism.^41^ Fig. 4a displays the current mismatch between top and bottom cells (Δ*J*_sc_) as a function of the thickness of each perovskite layer (*D*_1_ and *D*_2_, respectively), the white dotted line indicating the current-matching condition. For this calculation the thicknesses of the different non-active layers are kept constant (see Methods). Fig. 4b shows the *J*_sc_ across the contacts of the tandem device as a function of *D*_1_ and *D*_2_. It can be observed that the photogenerated current increases with *D*_1_. Notice that above *D*_1_ = 250 nm, *D*_2_ becomes significantly thicker to achieve current matching. For this reason, in order to show the experimental feasibility of the proposed architecture, the analysis that follows focuses on a perovskite-on-perovskite device in which *D*_1_ = 270 nm and *D*_2_ = 560 nm, which leads to *J*_sc_ = 17.20 mA cm^–2^. Notice that this value is comparable to those reported for actual devices.^38^ The open circuit voltages for the top and bottom cells are found to be 1.32 V and 0.92 V, respectively, according to the SQ theory, which would bring the voltage of the double-junction device to 2.24 V. Thus, the efficiency of such a perovskite-on-perovskite tandem device would reach 34% assuming a fill factor of 0.88. Fig. 4c displays the spectral dependence of the spatial distribution of the electric field intensity calculated inside the tandem cell. Bright and dark fringes arise from the interference between light reflected and transmitted by each layer of the multi-layered system. The calculations indicate that: (i) *λ* < 600 nm barely reaches the bottom cell; (ii) 600 nm < *λ* < 800 nm are absorbed by top and bottom cells simultaneously; and (iii) *λ* > 800 nm reaches the metallic contact of the cell. Indeed, light distribution across FTO, the hole transporting material, and gold suggests the presence of parasitic absorption. In order to assess this effect we estimate the EQE of the tandem cell. Fig. 4d displays the EQE of the tandem device together with the ones of the constituent cells. The total EQE remains over 85% for most of the spectral range until *λ* = 1100 nm that is the absorption onset of the CH_3_NH_3_Sn_0.85_Pb_0.15_I_3_ material. Notice that the EQE features a dip at *λ* = 875 nm, where the fraction of light absorbed by the perovskite absorbers reduces up to 54%, 39% of the incident light being reflected at the entrance of the cell (see Fig. S11 of the ESI†). It is also important to stress that the EQE gradually diminishes from *λ* = 950 nm because both FTO and spiro-OMeTAD, standard materials in Pb-based PSCs, absorb a non-negligible fraction of the incident light in the NIR.

In conclusion, we have demonstrated a novel synthesis route based on solvent engineering and anti-solvent deposition that enables the preparation of pin-hole free dense layers of CH_3_NH_3_Sn_*x*_Pb_1–*x*_I_3_, featuring perovskite films that are structurally and optically stable. This has allowed us to perform an in-depth optical characterization to achieve the complex refractive index of the different composition perovskite films. The performance of hypothetical solar cells based on these CH_3_NH_3_Sn_*x*_Pb_1–*x*_I_3_ light harvesters is estimated. Our results, based on realistic device parameters, indicate that fine tuning of the composition would eventually lead to significant enhancement of the power conversion efficiency of perovskite solar cells. In particular, adding 15% of Sn to the perovskite absorber results in a 25% increase of the photo-generated current. This may yield a 10% increase of the performance of state-of-the-art perovskite devices. The bandgap tunability and optical quality of the solution processed films herein reported may also allow the fabrication of perovskite-on-perovskite tandem solar cells of even superior efficiencies. Our proposal is intended to promote the use of perovskites that would give rise to considerable cost reductions in multi-junction technology while potentially featuring performances exceeding those theoretically achievable by single junction solar cells. These findings should encourage further research to achieve working devices of improved chemical stability that could surpass the performance of pure lead perovskite cells.

## Methods

### Fabrication

#### Synthesis of tin/lead-based perovskites

The perovskite films were prepared using SnI_2_ (1.2 M, 99.99% Aldrich) or PbI_2_ (1.2 M, TCI Chemicals) and MAI (1.2 M, Dyesol) in anhydrous DMF : DMSO 4 : 1 (v/v, Acros). We employed a mixture of two solvents with distinct degrees of solubility, *i.e.* DMF and DMSO, 1 : 4 DMSO : DMF (v/v) to dissolve the MAI/SnI_2_ and MAI/PbI_2_ precursors. Although DMF provides high solubility (concentrations up to 1.5 M can be achieved in MAI, PbI_2_ and SnI_2_ perovskite solutions), it typically yields Sn/Pb perovskite films with limited optical quality. The addition of DMSO to DMF-based perovskite precursor solutions gives rise to dense and smooth films, and represents a key factor to achieve high optical quality perovskite Sn/Pb films. We dissolved the metal iodides first in DMF at a concentration of 1.5 M. We then diluted the solution to 1.2 M by adding DMSO. Solutions of pure Sn and Pb perovskite precursors were pipetted in the right volumes in order to tune the Sn/Pb fraction in the mixes. Specifically, three mixes of 15%, 50% and 85% Sn were prepared. Apart from the metal, everything else (*i.e.* concentration, solvent mixture, *etc.*) was kept constant.

#### Film preparation

Glass substrates were first wiped with acetone, and then cleaned for 10 min in piranha solution (H_2_SO_4_/H_2_O_2_ = 3 : 1) followed by 10 min in a plasma cleaner. The perovskite solution was spin-coated in a two-step program, first at 1000 rpm for 10 s and then at 4000 rpm for 30 s. During the second step, 100 μL of chlorobenzene was poured on the spinning substrate 20 s prior to the end of the program. Such an anti-solvent deposition method induces fast crystallization, which has been proved before to enhance the smoothness of pure Pb based films.^48^ The substrates were then annealed at 100 °C for 1 h in a nitrogen filled glove box. The films were encapsulated with PMMA or with a glass slide adhered with epoxy resin. For PMMA encapsulation, 10 mg mL^–1^ of PMMA (Aldrich) was spin-coated on the perovskite films at 2000 rpm for 20 seconds. For glass encapsulation, a glass slide is positioned atop the perovskite film and a 2-component epoxy (Varian Torr Seal High Vacuum) is used around the edges making a proper seal. Encapsulation was made in a glovebox, immediately after perovskite film annealing and under very low oxygen concentration (<30 ppm) in order to prevent oxidation of the Sn-based perovskites.

#### Solar cell preparation

Fluorine doped tin oxide coated glass slides (Sigma-Aldrich, ∼7 Ω^–1^) were cleaned by sonication in 2% Hellmanex solution for 15 minutes. After rinsing with deionised water and ethanol the substrates were again sonicated with isopropanol and rinsed with acetone. The substrates were plasma treated for 5 minutes. 30 nm TiO_2_ compact layer was deposited on FTO *via* spray pyrolysis at 450 °C from a precursor solution of titanium diisopropoxide bis(acetylacetonate) in anhydrous ethanol. Mesoporous TiO_2_ was deposited by spin coating 30 nm particle paste (Dyesol 30 NR-D) diluted in ethanol for 10 s at 4000 rpm with a ramp of 2000 rpm  s^–1^, to achieve a 150 to 200 nm thick mesoporous layer. Perovskite films were cast from a precursor solution containing SnI_2_ (0.18 M, 99.99% Aldrich), PbI_2_ (1.02 M, TCI Chemicals) and MAI (1.2 M, Dyesol) in anhydrous DMF : DMSO 4 : 1 (v/v, Acros). The perovskite solution was spin-coated in a two-step program; first at 1000 rpm for 10 s and then at 4000 rpm for 30 s. During the second step, 100 μL of chlorobenzene was poured on the spinning substrate 25 s before the end of the program. The substrates were then annealed at 100 °C for 1 hour in a nitrogen glove box (<30 ppm O_2_). Subsequently, the substrates were cooled down to room temperature and the HTM layer was spun. Two HTM materials were employed: spiro-OMeTAD and PTAA. A spiro-OMeTAD (Luminescence Technology Corp.) solution (70 mM in chlorobenzene) doped with bis(trifluoromethylsulfonyl)imide lithium salt (Li-TFSI, Aldrich), tris(2-(1*H*-pyrazol-1-yl)-4-*tert*-butylpyridine)-cobalt(iii)tris(bis(trifluoromethylsulfonyl)imide) (FK209, Dyenamo) and 4-*tert*-butylpyridine (TBP, Aldrich) was spun at 4000 rpm for 20 s. The molar ratio of additives for spiro-OMeTAD was: 0.5, 0.03 and 3.3 for Li-TFSI, FK209 and TBP, respectively. Alternatively, in the case that PTAA was employed as the HTM, 10 mg of PTAA (EM INDEX) was dissolved in 1 mL of chlorobenzene, without any additives, and spin-coated at 4000 rpm for 20 s. Finally, 70 nm of gold was thermally evaporated under high vacuum on top of the device. All device preparation, including gold evaporation was done inside a glovebox to avoid the contact of the perovskite film with air.

### Characterization

#### Structural analysis

The crystal structure of the samples was analyzed by XRD using a Bruker D8 Advance X-ray diffractometer using Cu Kα radiation (*λ* = 0.154178 nm) at a scanning rate of 0.05° s^–1^ in the 2*θ* range from 10° to 60°. A ZEISS Merlin HR-SEM at 1 kV was used to characterize the morphology of the perovskite films. A scanning electron microscope Hitachi 5200 operating at 5 kV was used to take cross-section images.

#### Optical characterization

Direct measurements of the spectral dependence of the total and diffuse reflectance and transmittance, as well as absorptance of the perovskite thin films were carried out using an integrating sphere (Labsphere RTC-060-SF), a halogen lamp (Ocean Optics HL-2000) as the light source, and two spectrometers working in the visible (Ocean Optics USB 2000+) and the NIR (Ocean Optics NIRQuest 512) regions of the electromagnetic spectrum as detectors.

#### Solar cell characterization

For photovoltaic measurements, a solar simulator from ABET Technologies (Model 11016 Sun 2000) with a xenon arc lamp was used and the solar cell response was recorded using a Metrohm PGSTAT302N Autolab. The intensity of the solar simulator was calibrated to 100 mW cm^–2^ using a silicon reference cell from ReRa Solutions (KG5 filtered). *J*–*V* curves were measured in reverse and forward bias at a scan rate of 10 mV s^–1^. A 0.4 cm^2^ mask was used. Measurements were performed inside a glovebox to avoid the contact of the perovskite film with air.

### Theoretical modelling

#### Complex refractive index determination

We develop a model based on the transfer matrix method, which allows extracting the complex refractive index (*n* + i*k*) of our prepared CH_3_NH_3_Sn_*x*_Pb_1–*x*_I_3_ perovskite thin films by fitting a model dielectric function to the experimental reflectance and transmittance spectra. We calculate the dielectric function according to the Forouhi–Bloomer formulation.^15,49^ Full details are provided in the ESI.†

#### Shockley–Queisser theory

Voltage (*V*) dependence of the current (*I*) extracted from single junction solar cells based on light absorbing materials with different *E*_g_ can be calculated assuming that radiative recombination (RR) is the only source of electron–hole pair recombination*I*(*V*, *E*_g_) = *I*_ph_(*E*_g_) – RR(*V*, *E*_g_)

*I*_ph_ being the photogenerated current.
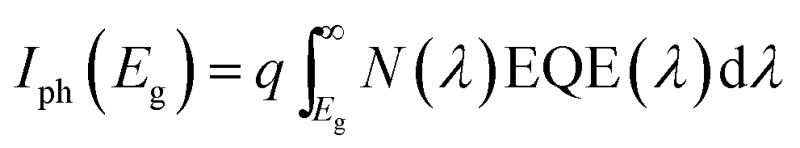
with *q* the electron charge, *λ* the wavelength of light, *N*(*λ*) the number of photons provided by the Sun at Air Mass 1.5, and EQE(*λ*) the external quantum efficiency. RR is given by
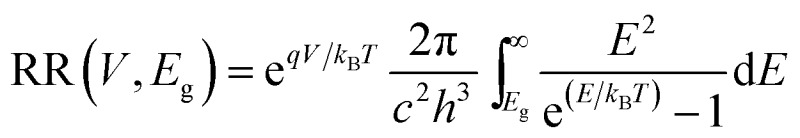
with *c* the speed of light, *h* the Planck constant and *k*_B_ the Boltzmann constant. *T* is the temperature and *E* is the energy.

#### Photogenerated current calculation

The ideal short circuit photocurrent density, *J*_sc_ = *I*(0, *E*_g_), is achieved when the EQE is 100% for every frequency above *E*_g_, which implies that all absorption, collection and injection efficiencies are considered to be 100%. A realistic estimation of the photocurrent can be obtained by using an optical model, in which the fraction of light absorbed by the active material can be accurately assessed. For a single junction device, we consider a 700 nm transparent conductive substrate (FTO) followed by an electron selective layer (a dense TiO_2_ film), a 50% porous scaffold made of TiO_2_ (85 nm) that is fully infiltrated by the perovskite, a 600 nm perovskite capping layer, a hole selective layer of 240 nm (spiro-OMeTAD) and a 50 nm gold contact. For a double junction device, we consider a 700 nm transparent conductive substrate (FTO), a 10 nm dense SnO_2_ film, 270 nm of CH_3_NH_3_PbI_3_, 240 nm of spiro-OMeTAD, 10 nm of denser SnO_2_ film, 560 nm of CH_3_NH_3_Sn_0.85_Pb_0.15_I_3_, 240 nm of spiro-OMeTAD and a 50 nm gold contact.

#### Power conversion efficiency (PCE) calculation

The PCE of a device is determined by
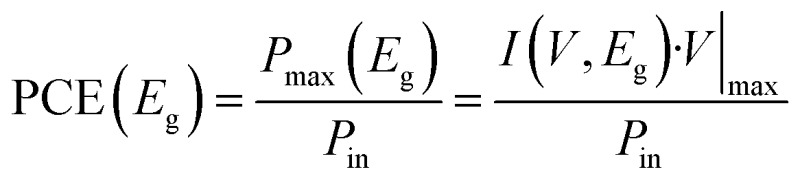
where *P*_max_ is the maximum power extracted from the cell and *P*_in_ is the incident sunlight power.
