# Supplementary material for: Optical analysis of CH3NH3SnxPb1–xI3 absorbers: a roadmap for perovskite-on-perovskite tandem solar cells
Source: J Mater Chem A Mater. 2016 Jun 29;4(29):11214–21. doi: 10.1039/c6ta04840d (PMC5059782; doi:10.1039/c6ta04840d)
Supplement: Supplementary file 1 [file TA-004-C6TA04840D-s001.pdf]

Electronic Supplementary Material (ESI) for Journal of Materials Chemistry A.
This journal is © The Royal Society of Chemistry 2016

Supporting information: Optical analysis of $CH_3NH_3Sn_xPb_{1-x}I_3$ absorbers: a roadmap for perovskite-on-perovskite tandem solar cells

Miguel Anaya,†[a] Juan P. Correa,†[b] Gabriel Lozano,[a] Michael Saliba,[c] Pablo Anguita,[a] Bart Roose,[d] Antonio Abate,[c] Ullrich Steiner,[d] Michael Grätzel,[c] Mauricio E. Calvo,[a] Anders Hagfeldt[b] and Hernán Míguez*[a]*

[a] Institute of Materials Science of Seville, Spanish National Research Council-University of Seville, Calle Américo Vespucio 49, 41092, Seville, Spain.

[b] Laboratory for Photomolecular Science, Institute of Chemical Sciences and Engineering, Ecole Polytechnique Fédérale de Lausanne, CH-1015-Lausanne, Switzerland.

[c] Laboratory for Photonics and Interfaces, Institute of Chemical Sciences and Engineering, Ecole Polytechnique Fédérale de Lausanne, CH-1015-Lausanne, Switzerland.

[d] Adolphe Merkle Institute, Chemin des Verdiers 4, CH-1700 Fribourg, Switzerland.

†These authors contributed equally to this work.

AUTHOR INFORMATION

Corresponding Authors

*h.miguez@csic.es

*juan.correa@epfl.ch

In order to characterize the samples outside a glovebox, $CH_3NH_3Sn_xPb_{1-x}I_3$ films were encapsulated using a PMMA protecting layer or a cover glass combined with an epoxy sealing. Figure S1 and S2 present XRD and light absorption measurements at various aging states of films encapsulated with the different procedures. The glass cover allows maintaining a N_2 atmosphere around the film, avoiding moisture contact with the perovskite material, resulting in samples that do not degrade over months.

FIGURE S1

XRD patterns of $CH_3NH_3SnI_3$ films aged for 2 weeks with different encapsulation procedures: standard PMMA protecting the perovskite layer (top diagram), and double glass sealed with epoxy (bottom diagram). While the perovskite structure is lost when the first method is employed, for the latter no degradation of the film is observed.

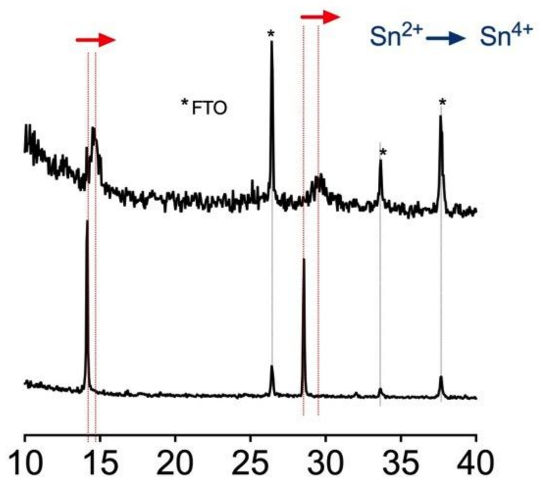

The crystal structures of compositions varying from 0 to 100% Sn were analyzed by XRD (Figure 1g). As shown by Hao et al.,[1] the two peaks between 2θ 22 and 25° which clearly indicate the tetragonal structure of the 0 and 15% Sn samples, disappear with Sn concentrations of 50% or above, where the structure moves towards the cubic. For all compositions two peaks are dominant in the pattern. One is located at just above 14° which can be indexed as (002) and (110) for the tetragonal I4cm space group and those samples with less than 50% Sn, and as (001) for the cubic P4mm for samples above 50% Sn. The second prominent peak is located at 28.5°, which can be indexed as (004) and (220) for the tetragonal (0 and 15% Sn) and (002) for the cubic (50, 85 and 100% Sn).[2] A gradual shift in both of the prominent peaks is detected as seen in Figure 1h and i, confirming what has been shown in similar studies.[1,3]

FIGURE S2

Experimental total absorptance spectra of $CH_3NH_3Sn_{0.5}Pb_{0.5}I_3$ films aged for 4 weeks with the two encapsulation procedures: standard PMMA (left panel), and double glass sealed with epoxy (right panel). Black solid lines represent fresh films whose optical response was measured immediately after deposition. Grey dashed lines correspond to fimls expose to air for 4 weeks. While the optical response expected for this kind of perovskite structure is lost with time when the first encapsulation method is employed, for the latter no degradation of the film is taken place.

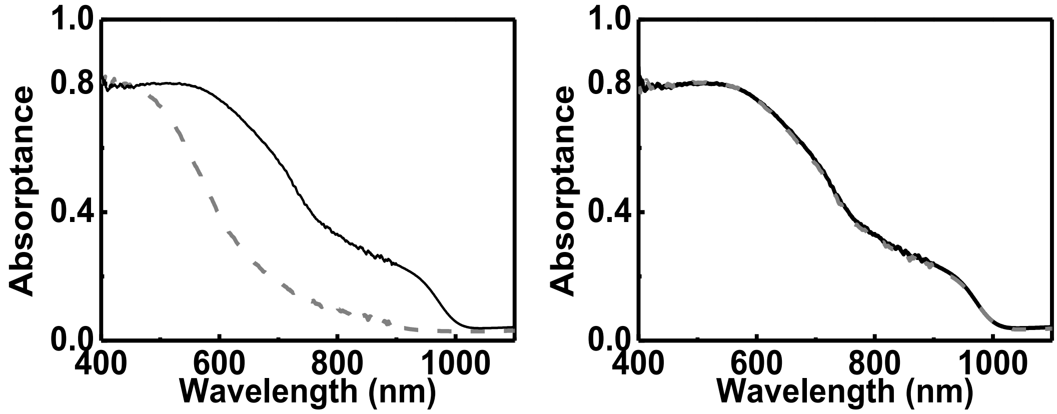

FIGURE S3

Experimental diffuse reflectance (left panel) and transmittance (right panel) for perovskite films in which the percentage of Sn is increase at the expense of Pb: 0% Sn (black solid line), 15% Sn (dark grey solid line), 50% Sn (grey solid line), 85% Sn (black dashed line) and 100% Sn (dark grey dashed line).

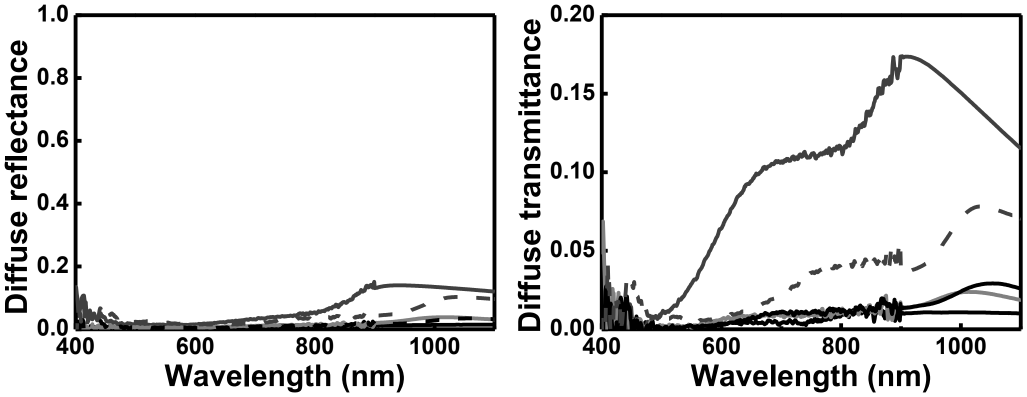

Structural analysis performed by SEM reveals the smoothness of the different perovskite films. This homogeneity together with the big perovskite grains, that the films present, give rise to the low diffuse reflectance that we measured employing an integrating sphere.

FIGURE S4: Experimental absorptance of perovskite films in which the percentage of Sn is increased at the expense of Pb: 0% Sn (blue line), 15% Sn (blue-green line), 50% Sn (green line), 85% Sn (orange line) and 100% Sn (red line).

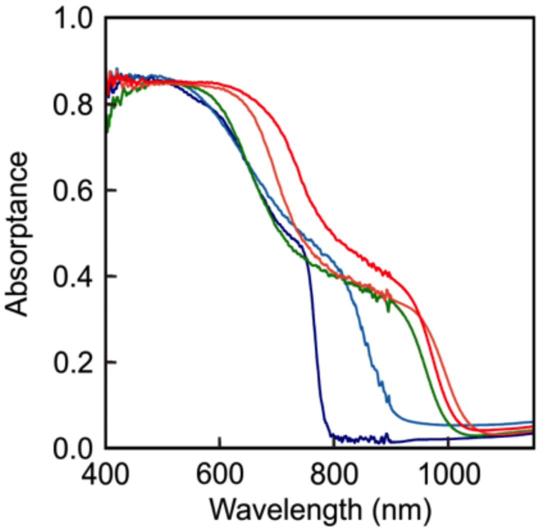

The band gap of the pure lead perovskite is found at Eg=1.60 eV. As the fraction of Sn in the precursor solution increases, the band edge shifts to longer wavelengths, reaching a minimum value in energy, Eg = 1.17 eV, when the Sn content is 85%. After this monotonous redshift, the band gap energy increases, reaching a value of Eg = 1.21 eV for the pure Sn-based perovskite (see Figure 2b). Such band gap evolution, already discussed elsewhere, originates from a difference in the spin-orbit coupling and a crystal phase change from tetragonal to cubic as XRD analysis presented in Figure 1g indicates.

FIGURE S5: Real (grey) and imaginary (black) parts of the complex dielectric constant of the different mixed metal perovskite structures studied: (a) $CH_3NH_3SPbI_3$, (b) $CH_3NH_3Sn_{0.15}Pb_{0.85}I_3$, (c) $CH_3NH_3Sn_{0.50}Pb_{0.50}I_3$, (d) $CH_3NH_3Sn_{0.85}Pb_{0.15}I_3$, and (e) $CH_3NH_3SnI_3$.

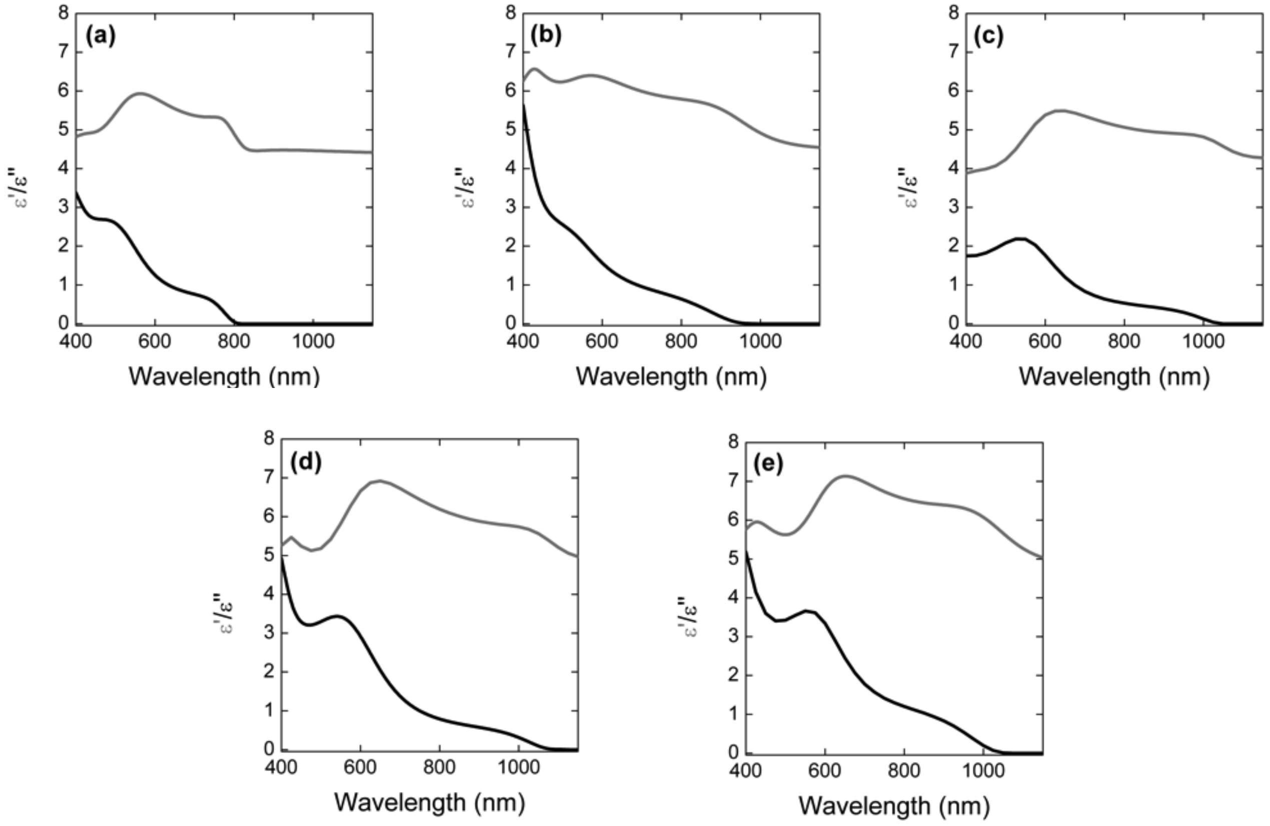

TABLE 1

We modelled the perovskite film considering air as the incoming and outgoing media and three layers of different thicknesses: i) a 1mm-thick glass substrate that is considered to be incoherent, ii) a perovskite film of thickness d_{bulk}, and iii) a very thin layer of thickness d_{rough} consisting of perovskite and air to account for the surface roughness of the perovskite film. We use a Forouhi-Bloomer model in the parametrization of Jobin Yvon that consists of 3 oscillators (with E_i, f_i, Γ_i the position, strength and width of each oscillator, respectively), which has been already employed to account for the spectral dependence of the complex refractive index elsewhere.[4] Model parameters attained for the different Sn/Pb perovskites are displayed in Table 1. The energy band gap (Eg) and d_{bulk} are obtained experimentally, and therefore they are not considered as fitting parameters in the model. The term $n\infty$ corresponds to the value of the refractive index when $E\rightarrow\infty$.

$CH_3NH_3Sn_xPb_{1-x}I_3$

Model parameters	0%	15%	50%	85%	100%
**Eg (eV)**	1.55	1.28	1.18	1.140	1.170
**n_∞**	1.954	1.95	1.95	1.950	1.956
**f_1**	0.166	0.186	0.106	0.126	0.224
**f_2**	0.092	0.053	0.074	0.11	0.072
**f_3**	0.015	0.034	0.01	0.017	0.050
**E_1 (eV)**	1.552	1.293	1.18	1.143	1.189
**E_2 (eV)**	2.345	2.258	2.129	2.089	2.074
**E_3 (eV)**	3.304	3.303	3.3	3.3	3.370
**Γ_1 (eV)**	0.064	0.199	0.1	0.1	0.159
**Γ_2 (eV)**	0.446	0.493	0.463	0.475	0.392
**Γ_3 (eV)**	0.301	0.352	0.6	0.301	0.513
**d_{bulk} (nm)**	250	240	240	240	240
**d_{rough} (nm)**	20	20	1	10	16

FIGURE S6: Real (grey) and imaginary (black) parts of the complex refractive index of the different components of the perovskite solar cell: (a) FTO,[5] (b) TiO_2,[6] (c) spiro-OMeTAD,[7] and (d) gold.[8] The optical constants for the glass substrate are considered constants, being n=1.51 and k=0.

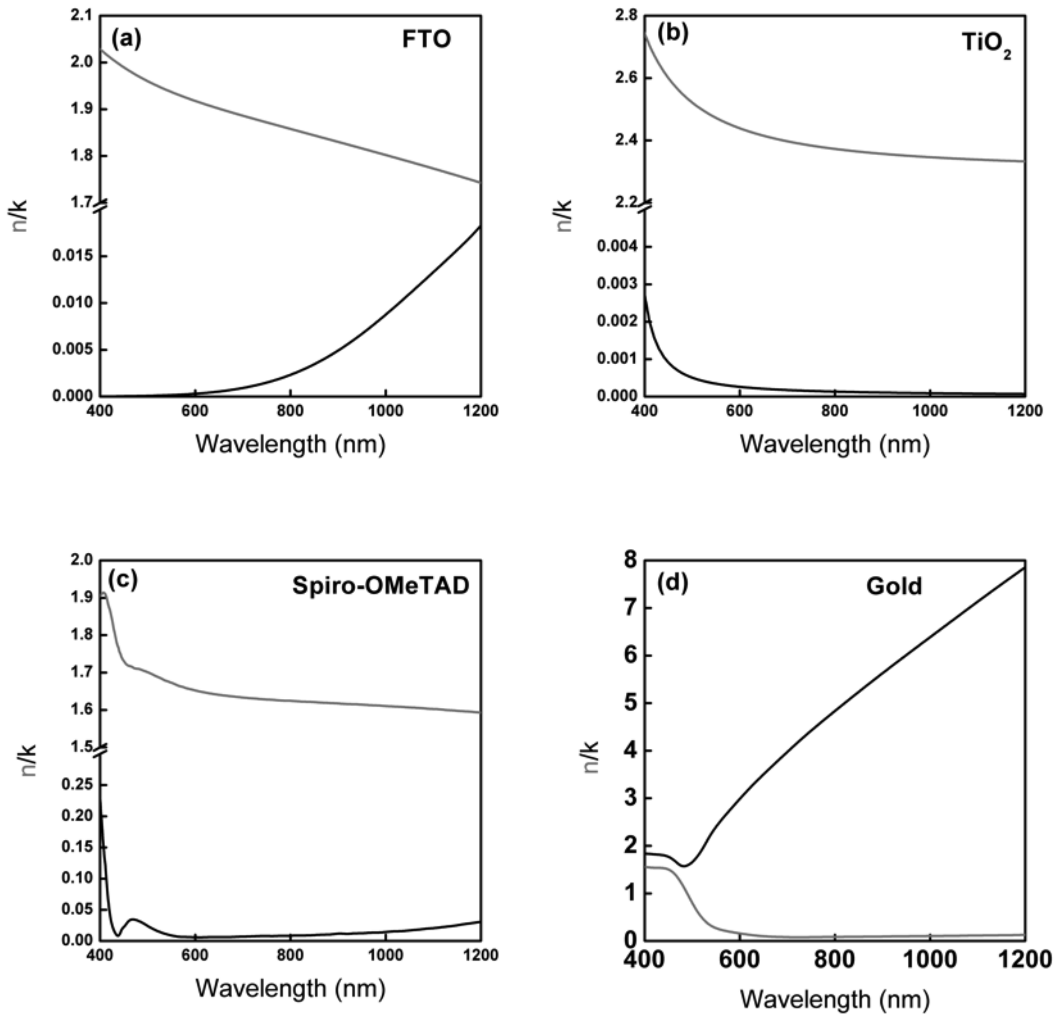

FIGURE S7: Calculated maximum short circuit current (Jsc) that an ideal $CH_3NH_3Sn_xPb_{1-x}I_3$-based solar cell would have depending on the thickness of the absorber layer capping layer: 0% Sn (blue line). 15% Sn (blue-green line). 50% Sn (green line). 85% Sn (orange line) and 100% Sn (red line). It can be noted that for all the different perovskite compositions Jsc slightly oscillates when increasing perovskite film thickness due to light interference phenomena. Moreover. Jsc almost saturates for perovskite capping layer thicknesses above 600 nm for all the considered compositions.

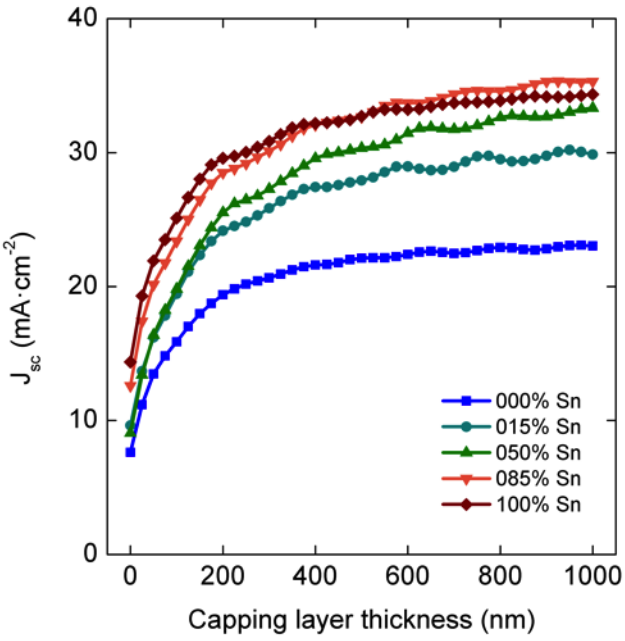

We prepared devices of $CH_3NH_3PbI_3$ and $CH_3NH_3Sn_{0.15}Pb_{0.85}I_3$, as shown in Figure S8. The unoptimized device architecture of FTO/compact-TiO2/mesoporous-TiO2/Perovskite/HTM/gold was used for this study, as seen in the cross-sectional SEM in Figure S8a (Photographs of films and devices are displayed in Figure S9). Remarkably, it was found that careful selection of the hole selective layers used (and their dopants), played an important role in the performance, and, presumably, in the degradation mechanism associated with this interface. Figure S8b shows the performance of a $CH_3NH_3Sn_{0.15}Pb_{0.85}I_3$ device using spiro-OMeTAD with dopants compared to polytriarylamine polymer (PTAA) without any additives. Devices using doped spiro-OMeTAD showed a severe decrease in the photocurrent respect to the undoped PTAA analogue, i.e. ~4 $mA\cdot cm^{-2}$ vs 25 $mA\cdot cm^{-2}$. Since spiro-OMeTAD is well known to require additives (or alternatively oxygen, causing oxidation of the molecule) for improving its mobility,[9,10] PTAA was used instead, which has been shown to perform rather well without additives.[11] Indeed, devices with PTAA and no additives performed much better than those with Spiro-OMeTAD and no additives. We attribute this effect to the oxidization of the Sn^{2+} to Sn^{4+} by the Co, 4-tert-Butylpyridine (TBP), and Li additives used to obtain the highest photovoltaic performance. PTAA with no additives was therefore used as the HTM in order to show the potential of the current of the devices. Figure S8b shows that devices using $CH_3NH_3Sn_{0.15}Pb_{0.85}I_3$ as the absorber yields substantially higher photocurrents (25 $mA\cdot cm^{-2}$) than those of the pure Pb analogues (20 $mA\cdot cm^{-2}$). The Sn-Pb alloy also displays relatively high voltages comparable to those reported by others.[12] FF for devices containing both compounds show low values, however, we note that this is proof-of-concept experiment to justify our trends in the currents predicted.

FIGURE S8. (a) Scanning electron micrograph of a perovskite solar cell in which a layer of $CH_3NH_3Sn_{0.15}Pb_{0.85}I_3$ acts as absorber. (b) Current–voltage (*JV*) characteristics of a $CH_3NH_3Sn_{0.15}Pb_{0.85}I_3$ solar cell in which PTAA (red curve) and spiro-OMeTAD (black) is employed as hole transporting material (HTM). The blue curve shows the *JV* characteristics of a $CH_3NH_3PbI_3$ in which PTAA acts as HTM.

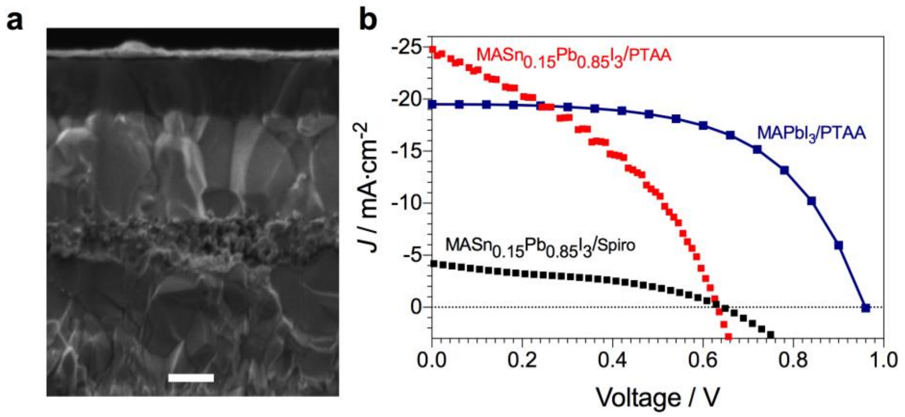

TABLE 2. Photovoltaic parameters of the different cells fabricated.

	J_{sc} (mA·cm^{-2})	V_{oc} (V)	*FF* (%)	*PCE* (%)
$CH_3NH_3PbI_3$/PTAA	19.5	0.960	58.2	10.9
$CH_3NH_3Sn_{0.15}Pb_{0.85}I_3$/PTAA	24.8	0.633	38.9	6.1
$CH_3NH_3Sn_{0.15}Pb_{0.85}I_3$/spiro	4.2	0.644	38.1	1.0

FIGURE S9: Photograph of a-b. devices, c-d perovskite films as seen from the top and e-f as seen from the glass side. a, c and e are pure Pb perovskite whereas b, d and f contain 15% Sn. No visible (color change) degradation is seen in the films or devices when exposed to air after 3 days.

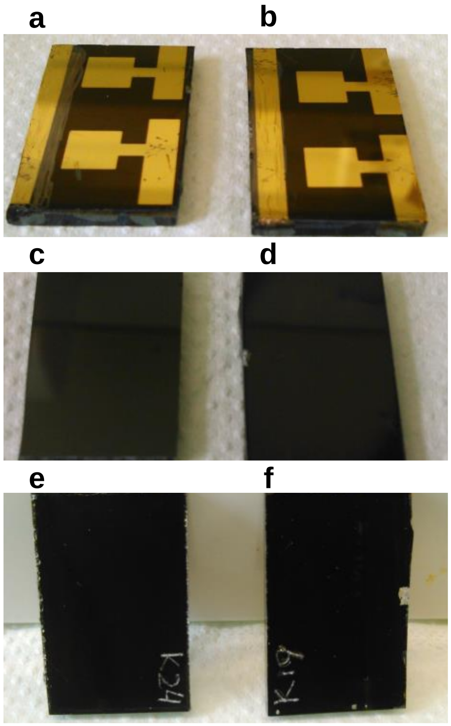

FIGURE S10: (a) Difference between short circuit currents extracted from the top and bottom cells composing a tandem device with mesostructured architecture, in which the $CH_3NH_3PbI_3$ (D_1) and $CH_3NH_3Sn_{0.85}Pb_{0.15}I_3$ (D_2) thicknesses are varied. White dashed line indicates the particular thicknesses for which the current matching is attained. Inset shows the architecture of the simulated device: 1 mm of glass substrate (1), 650 nm of FTO layer (2), 50 nm of TiO_2 compact layer (3), 85 nm of 50% porous TiO_2 scaffold fully infiltrated by $CH_3NH_3PbI_3$ (4), D_1 nm of $CH_3NH_3PbI_3$ capping layer (5), 240 nm of spiro-OMeTAD (6), 50 nm of TiO_2 compact layer (3), 85 nm of 50% porous TiO_2 scaffold fully infiltrated by $CH_3NH_3PbI_3$ (4), D_2 nm of $CH_3NH_3Sn_{0.85}Pb_{0.15}I_3$ capping layer (5), 240 nm of spiro-OMeTAD (6), and 50 nm of gold contact (7). (b) Matched short circuit current values for each pair of D_1-D_2 thicknesses. Grey mark points out the configuration that is more deeply analysed in the next panels, in which D_1=300 nm and D_2=1000 nm. (c) Calculated spatial (y-axis) and spectral (x-axis) distribution of the electric field intensity enhancement along the cross section of the selected perovskite-on-perovskite tandem solar cell. White dashed lines specify the interfaces between the different layers. (d) Calculated external quantum efficiency corresponding to the top (black line) and bottom (grey line) cells.

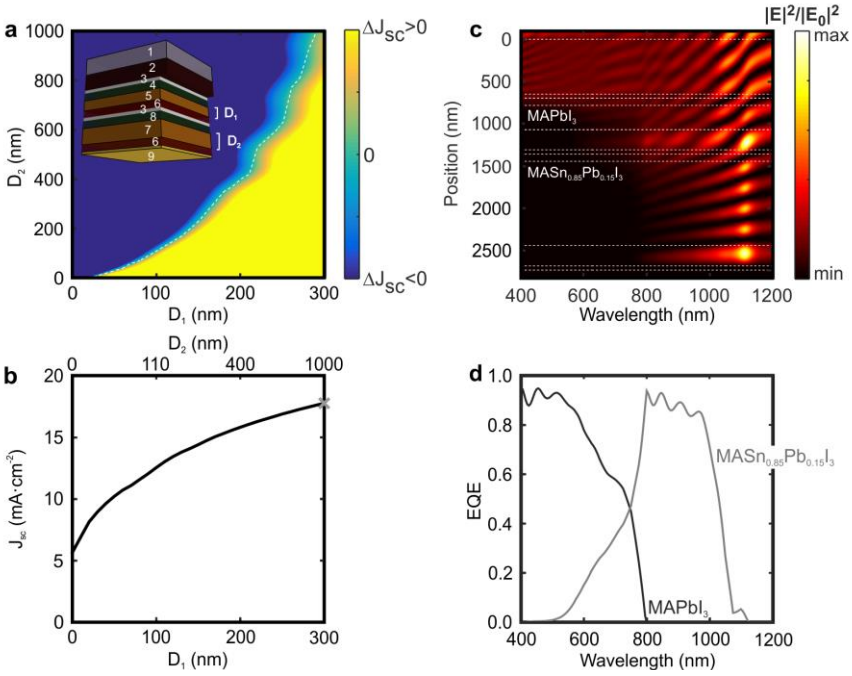

FIGURE S11: Spectral dependence of the reflectance (blue), absorptance (black) of the perovskite-on-perovskite tandem device shown in Figure 4a of the main part of the manuscript. Spectral dependence of the fraction of the incident light absorbed by the spiro-OMeTAD (red), FTO (grey), and Au (orange).

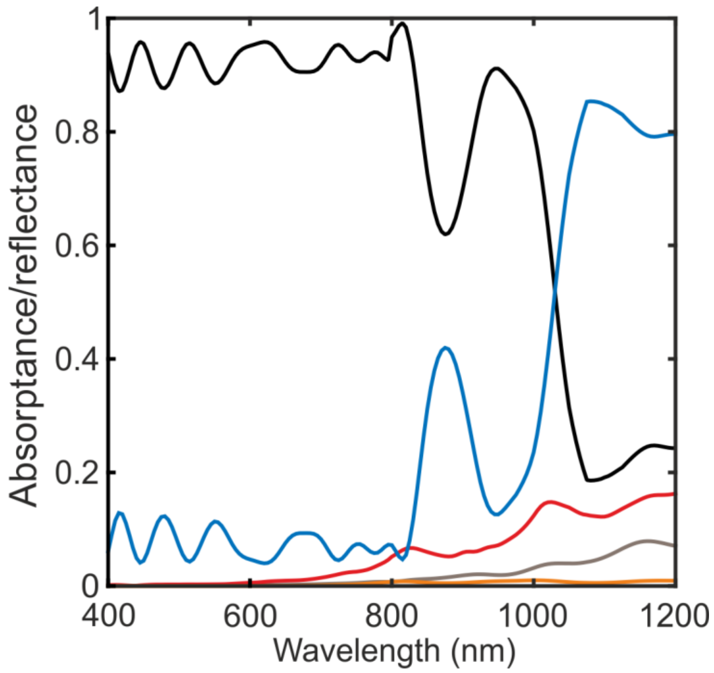

References

1 F. Hao, C. C. Stoumpos, R. P. H. Chang and M. G. Kanatzidis, *J. Am. Chem. Soc.* 2014, **136**, 8094.

2 T. J. Jacobsson, L. J. Schwan, M. Ottosson, A. Hagfeldt and T. Edvinsson, *Inorg. Chem.*, 2015, **54**, 10678.

3 Y. Ogomi, A. Morita, S. Tsukamoto, T. Saitho, N. Fujikawa, Q. Shen, T. Toyoda, K. Yoshino, S. S. Pandey and T. Ma, *J. Phys. Chem. Lett.*, 2014, **5**, 1004.

4 P. Löper, M. Stuckelberger, B. Niesen, J. Werner, M. Filipič, S.-J. Moon, J.-H. Yum, M. Topič, S. De Wolf and C. Ballif, *J. Phys. Chem. Lett.* 2015, **6**, 66.

5 S. Wenger, M. Schmid, G. Rothenberger, A. Gentsch, M. Grätzel and J. O. Schumacher, *J. Phys. Chem. C* 2011, **115**, 10218.

6 J. R. Devore, *J. Opt. Soc. Am*. 1951, **41**, 416.

7 M. Filipič, P. Löper, B. Niesen, S. De Wolf, J. Krč, C. Ballif and M. Topič, *Opt. Express* 2015, **23**, A263.

8 S. Babar and J. H. Weaver, *Applied Optics* 2015, **54**, 477.

9 A. Abate, T. Leijtens, S. Pathak, J. Teuscher, R. Avolio, M. E. Errico, J. Kirkpatrik, J. M. Ball, P. Docampo, I. McPherson and H. J. Snaith, *Phys. Chem. Chem. Phys*. 2013, **15**, 2572.

10 A. Abate, D. R. Staff, D. J. Hollman, H. J. Snaith and A. B. Walker, *Phys. Chem. Chem. Phys*. 2014, **16**, 1132.

11 G.-W. Kim, G. Kang, J. Kim, G.-Y. Lee, H. I. Kim, L. Pyeon, J. Lee and T. Park, *Energy Environ. Sci*. 2016, DOI: 10.1039/C6EE00709K.

12 F. Zuo, S. T. Williams, P.-W. Liang, C.-C. Chueh, C.-Y. Liao and A. K.-Y. Jen, *Adv. Mater.* 2014, **26**, 6454.
